# Do Surface Slope and Posture Influence Lower Extremity Joint Kinetics during Cycling?

**DOI:** 10.3390/ijerph17082846

**Published:** 2020-04-21

**Authors:** Yunqi Tang, Donghai Wang, Yong Wang, Keyi Yin, Cui Zhang, Limin Zou, Yu Liu

**Affiliations:** 1 Key Laboratory of Exercise and Health Sciences of the Ministry of Education, Shanghai University of Sport, Shanghai 200438, China; 1510302010@sus.edu.cn (Y.T.); jason_wangdh@163.com (D.W.); bihai_zhixin@163.com (Y.W.); yky0504@outlook.com (K.Y.); 1510302016@sus.edu.cn (C.Z.); 9920020057@jgsu.edu.cn (L.Z.); 2 College of Art & Design, Shaanxi University of Science & Technology, Xi’an 710021, China; 3 Shanghai R&D Center, Lifesense Medical Electronics Co., Ltd., Shanghai 200051, China; 4 Department of Physical education, Liaocheng University, Liaocheng 252059, China; 5 Sport biomechanics lab, Shandong Institute of Sports Science, Jinan 250102, China; 6 Department of Physical education, Jinggangshan University, Ji’an 343009, China

**Keywords:** cycling, lower extremity, joint kinetics, surface slope, posture, joint function

## Abstract

The purpose of this study was to investigate the effects of surface slope and body posture (i.e., seated and standing) on lower extremity joint kinetics during cycling. Fourteen participants cycled at 250 watts power in three cycling conditions: level seated, uphill seated and uphill standing at a 14% slope. A motion analysis system and custom instrumented pedal were used to collect the data of fifteen consecutive cycles of kinematics and pedal reaction force. One crank cycle was equally divided into four phases (90° for each phase). A two-factor repeated measures MANOVA was used to examine the effects of the slope and posture on the selected variables. Results showed that both slope and posture influenced joint moments and mechanical work in the hip, knee and ankle joints (*p* < 0.05). Specifically, the relative contribution of the knee joint to the total mechanical work increased when the body posture changed from a seated position to a standing position. In conclusion, both surface slope and body posture significantly influenced the lower extremity joint kinetics during cycling. Besides the hip joint, the knee joint also played the role as the power source during uphill standing cycling in the early downstroke phase. Therefore, adopting a standing posture for more power output during uphill cycling is recommended, but not for long periods, in view of the risk of knee injury.

## 1. Introduction

Physical inactivity represents the fourth leading risk factor, with the highest epidemiological impact on population health worldwide [1]. Cycling, as one of the most popular physical fitness activities, meets the recommended levels of physical activity for populations and brings benefits to people of different ages [2]. In children and adolescents, cardiorespiratory endurance, muscular fitness, favorable body composition and bone health are all improved. In adults, the risk of early death, heart disease, stroke, type-II diabetes, high blood pressure, adverse blood lipid profiles, and metabolic syndrome are lower; cardiorespiratory and muscular fitness is also improved. In older adults, cognitive function is improved [3]. Acquiring an injury as a result of cycling, however, cannot be neglected; this is normally related to training intensity, load, or posture [4,5]. The intensity of cycling is strongly associated with changes in position, cadence, and power [6]. Therefore, the effect of cycling training depends on the abovementioned parameters that the cyclist chooses. A biomechanical study on cycling could provide people with more knowledge to improve cycling performance and prevent injuries.

In the field of biomechanics, researchers studied kinematics, kinetics and EMG to explore motion control mechanics and improve cycling performance [7]. Most of these studies focused on the effects of changing workload [8], pedaling cadence [8,9], power output [10,11,12,13,14,15,16], EMG [17,18,19,20,21,22,23] and gross efficiency [24,25,26] when cyclists were riding on level terrain, while there was much less research focused on cycling in either uphill or downhill conditions. Cycling on a surface with different slopes could help us to examine how the central nervous system (CNS) responds to different tasks in the musculoskeletal system, by monitoring muscle activity, joint moments, or works. From the perspective of races, the hill-climbing ability of a cyclist always plays a vital role in a long-distance competitive road race (i.e., Tour de France) [27,28]. The reasons why cyclists choose various postural strategies (seated or standing), to overcome higher loads and fatigue during hill-climbing, have elicited interest from many researchers. Therefore, cycling on different slopes with different postures could serve as an excellent model to explore the interaction effect between the task and environment in human lower extremity motion control.

The inherent control mechanism of the biological system is responsive to the environmental change with which the system interacts or the task the system has to perform [7]. Many researchers have investigated cycling performance with an inclined surface. Bertucci et al. [29] reported that the crank moment was very similar between level and uphill seated cycling. The results of Caldwell et al. [30] also showed no significant difference between level and uphill seated cycling in pedal force and crank moment. No significant effect in joint moments, with a modest increase in magnitude, was observed when changing the slope from 0% to 8% [31]. Actually, altering the slope in the cycling motion would cause lower extremities kinematics, kinetics, and muscle activity to change [32]. Why was no significant slope effect on joints moments observed? Perhaps this is because they just used the mean value parameters of the entire cycle to reflect the effect of slope on joints moments, and their interpretation may be confused when attempting to compare the different slopes.

Another interesting aspect is that cyclists used various postures to overcome high workload and to prevent fatigue during uphill climbing. In the aspect of cycling tasks, altering postures from a seated to a standing position perhaps dramatically changed the joint moments and mechanical work due to changes in the geometry of body segments during cycling [7]. Several studies discussed the cycling posture effect during uphill by crank moment, pedal force, and cycling efficiency [29,33,34]. Only a few studies have reported joint moments and power profiles produced during uphill cycling [30,35,36]. However, just analyzing the crank moments and joint mechanical work unilaterally in one crank cycle could not systematically explain whether changing posture would affect the human lower extremity joint kinetics. 

The studies mentioned above have provided much insight into the biomechanical changes in terms of body posture or surface slope on the lower extremity. Consequently, it would help us to explain the effect of changing slope and posture on human lower extremity motion and final cycling performance by analyzing joint moments and mechanical work [29,30,37,38,39]. However, previous research only analyzed integral, peak, or mean joint moments or mechanical work in one cycle, which would neglect some useful information. Notably, some joint moments changed their functional role, or the joint power output varied from producing to absorbing energy several times in one cycle. Therefore, dividing one crank cycle into several phases in detail could provide new insights to systematically understand the motion control mechanism of the lower extremity joints during cycling under different conditions.

To the best of our knowledge, the influence of slope and posture on lower limb kinetics during road cycling has not been investigated. Therefore, the overall purpose of the present study was to compare kinetics during cycling at different surface slopes and with different postures under the same power output of 250 watts (W). Firstly, it was hypothesized that the surface slope would significantly alter the lower extremity joint moments and mechanical work, particularly in the downstroke phase. The second hypothesis was that the lower extremity joint function would change with varying body postures during cycling.

## 2. Materials and Methods 

### 2.1. Participants

Seven male and seven female young professional cyclists (age: 15.1 ± 0.8 years, height: 171.9 ± 8.0 cm, body mass: 61.3 ± 7.3 kg, training experience: 2.9 ± 1.0 years) volunteered to participate in this study. All the participants were free of lower extremity musculoskeletal injuries for at least six months prior to the test and volunteered to participate in this study. All participants had competitive cycling experience. Each participant was asked to read and sign an informed consent. The project was approved by the Human Ethics Committee of the local university (2016037).

### 2.2. Instrumentation

**Figure 1:**
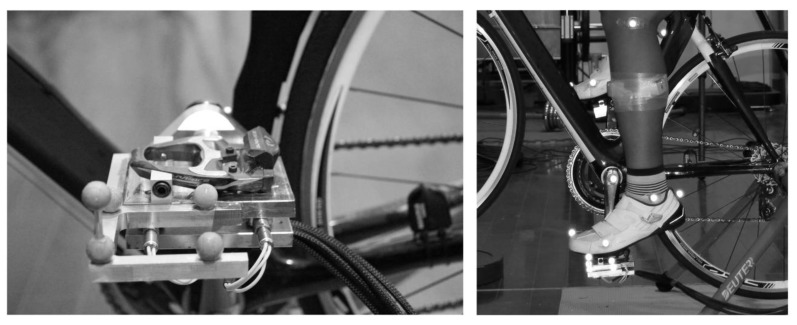
Image of the instrumented pedal force system with cleat fixed on the pedal.

A ten-camera motion analysis system (Vicon Motion Analysis Inc., Oxford, UK) was used to collect three-dimensional (3D) kinematic data. During the cycling trail, 40 reflective anatomical markers were placed on the participants’ anatomic landmarks, on the basis of the marker setup used in our earlier study [40]. Four pedal tracking markers were placed on the left side pedal, and a crank tracking marker was placed on the crank axis of the left cranks in order to identify crank cycles and to define the complete cycle with consecutive occurrences of crank top-dead-center (TDC), with the crank arm vertical and the pedal at its highest position. The bicycle was fitted with an SRM Science crank dynamometer system (Schoberer Rad Messtechnik, Julich, Germany), for sampling (1 Hz) of power output and pedal rate. A customized bike pedal (Figure 1), instrumented with four 3D force sensors (Type 9016C4, Kistler, Switzerland), coupled with one industrial charge amplifier (Type 9865E, Kistler, Switzerland), was placed on the left side of the pedal to measure 3D forces and moments (at 1200 Hz). A dummy pedal with the same mass and design was used to balance the instrumented pedal on the left side. This pedal force measurement system has been previously validated by Wang et al. [41].

### 2.3. Protocol

All tests were completed at the Sports Performance Research Centre of Shanghai University of Sport (Shanghai, China). The participants rode their bicycle (Scott Inc., Givisiez, Switzerland) on a Kinetic Road Machine Fluid Bike Trainer (Kinetic by Kurt Inc., Minneapolis, MN, USA), at a constant power output of 250 W. The power output and the pedal rate were monitored by SRM training system (Schoberer Rad Messtechnik Wireless Training System-SRAM Road GXP, 0.5% accuracy, Juelich, Germany). The validity of the SRM has been previously shown by Martin et al. [42] In order to minimize the effect of the shoe on cycling performance, all participants wore standard cycling shoes (TORBAL, Shimano Inc., Sakai, Osaka, Japan) with their suitable sizes. Meanwhile, the foot was firmly fixed with the pedal via a cleat, to ensure that there was no foot displacement with respect to the pedal during the tests. Seat height was adjusted such that the distance between the seat and the crank center was 100% of the participant’s greater trochanter length at a standing posture [43], to minimize seat height effects. Three cycling conditions consisted of level seated (LS), uphill seated (US) and uphill standing (ST) at an inclination of 14% slope, respectively. The 14% slope was the challenge surface inclination in the Tour de France. At the same time, participants maintained the instructed cadences by using a cadence monitor attached to the SRM training system. The cadences for the LS, US, and ST conditions were 65 revolutions per minute (RPM). If the average power output and pedaling cadence during the test were not within ±10 W and ±2 rpm of the desired instructions, respectively, then the test was failed and another test was performed.

Participants completed a warm-up session consisting of stretching and 5 min of cycling on Monark Ergometer (828E, Monark, Sweden) at a self-selected cadence, to familiarize themselves with the experimental conditions. Afterwards, the cycling condition order for each participant was randomized to minimize possible order effects. In each condition, the participants pedaled for approximately 2 min to establish a steady state, before data collection of 15 consecutive crank cycles. Participants were given sufficient time (a minimum of two minutes) to rest between each condition.

### 2.4. Data Processing

The raw kinematic and kinetic data were filtered using a low-pass fourth order Butterworth filter with zero lag, at a cutoff frequency of 4 Hz and 10 Hz, respectively [44]. One crank cycle was divided into four phases, each phase ranging 90° according to the crank angle (i.e., P1, 0° to 90°; P2, 90° to 180°; P3, 180° to 270°; P4, 270° to 360°), in which the top-dead-center point (TDC) was defined as 0°.

Inverse dynamics were used to calculate the joint moment and joint power with Visual3D (C-Motion Inc., Germantown, MD, USA), for a linked system of rigid segments; thigh, leg, and foot. All raw kinematic and kinetic data were imported into Visual3D software to process pedal reaction forces (PRFs) and lower extremity joint kinetics. The PRFs were calculated according to the vertical, anteroposterior and mediolateral components of the 3D force sensors. The moments were calculated based on the PRFs and distance measured between the four force sensors and of the pedal. The center of pressure (COP) was calculated according to the equations provided by the manufacturer. Then, the calculated PRFs, moments, and COP were converted into the lab coordinate system for inverse dynamics calculations. The right-hand rule was used to determine the polarity of the joint angles and joint kinetic data. The mean joint moment value in each phase was calculated by Excel 2010 (Microsoft Inc., Redmond, WA, USA). Positive values of ankle, knee and hip joint moment were defined as plantar flexion for ankle joint moment, and extension for knee and hip joint moment. In this study, the joint moment variables were not normalized with the participant’s body weight, because the majority of the participants’ weights were carried by the seat and handlebars. Net joint mechanical work was calculated by integrating the joint power with respect to time, and relative contributions of the ankle, knee, and hip joint were calculated by the percentage of total mechanical work (TMW) at the hip, knee, and ankle joints [38]. Data analyses of mechanical work were conducted following the codes developed by software Matlab 2016a (Matlab, Mathworks Inc., Natick, MA, USA).

### 2.5. Statistical Analysis

A descriptive statistical analysis was conducted on the demographic variables to describe the study population. To address the study hypothesis, a two-factor repeated measures MANOVA was analyzed to examine whether cycling conditions and phases and their interaction have a significant effect on the seven kinetic variables (i.e., crank moment, and moments and mechanical work of the ankle joint, knee joint, and hip joint moment). Follow-up univariate ANOVAs were conducted on each dependent variable and this was followed by pairwise comparisons with a Bonferroni correction, that compared the effect of the slope and posture across the four phases of cycle. A type I error rate less than 0.05 was chosen as the indication of statistical significance. All data analyses were performed using the SPSS statistical package, version 20.0 (IBM Corporation, Armonk, NY, USA).

## 3. Results

### 3.1. Joint Moments

**Figure 2:**
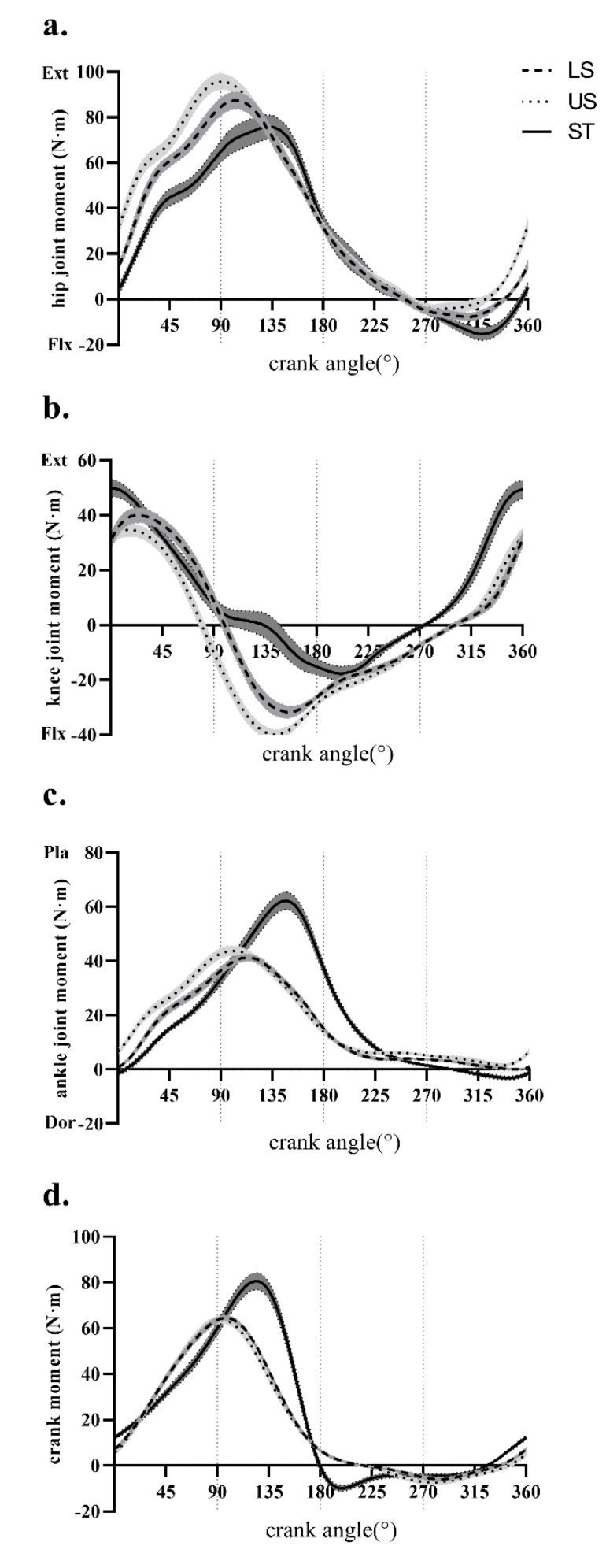
Average curves of the (**a**) hip joint, (**b**) knee joint, (**c**) ankle joint moments and the (**d**) crank moment (±SEM) in level seated (LS), uphill seated (US) and uphill standing (ST) conditions at a constant power output; (**a**) hip joint moment; (**b**) Knee joint moment; (**c**) ankle joint moment; (**d**) crank moment; Dor, Dorsiflexion; Ext, extension; Flx, flexion; Pla, plantarflexion; The vertical dash line in 90°, 180° and 270° refers to the border between each adjacent phase from P1 to P4.

The average curves (±SEM) for the three joint moments and the crank moment throughout one crank cycle in LS, US, and ST conditions are presented in Figure 2. Mean moments (±SD) of the hip, knee, and ankle joints and the crank from P1 to P4 in LS, US, and ST conditions are shown in Table 1. Repeated measures MANOVA of the lower extremity joints and crank moment in different cycling phases revealed a significant interaction between cycling conditions and phases (Wilks’ lambda = 0.004, F_24, 262.854_ = 42.915, *p* < 0.0001). Based on the MANOVA results, the following paragraphs detail the post-hoc ANOVA and pairwise comparison results for the hip, knee, and ankle joint moments and the crank moment in different phases with changing cycling slopes (LS vs. US) and postures (US vs. ST). 

There was a significant difference in joint moments between LS and US conditions in the hip joint (P1: *p* = 0.0005; P4: *p* = 0.0052), knee joint (P1: *p* = 0.0004; P2: *p* < 0.0001), and ankle joint (P1: *p* < 0.0001; P3: *p* = 0.0111; P4: *p* = 0.0240). The hip extension moment in the US condition increased in P1 compared to the LS condition. However, the hip moment changed from flexion to extension when the surface slope changed from LS to US conditions in P4 (*p* = 0.0052). Compared to the LS condition, the knee joint in the US condition showed a decreased extension moment in P1 (*p* = 0.0004), and an increased flexion moment in P2 (*p* < 0.0001). The ankle joint in the US condition showed an increased plantarflexion moment in P1, P3 and P4. 

**Figure 3:**
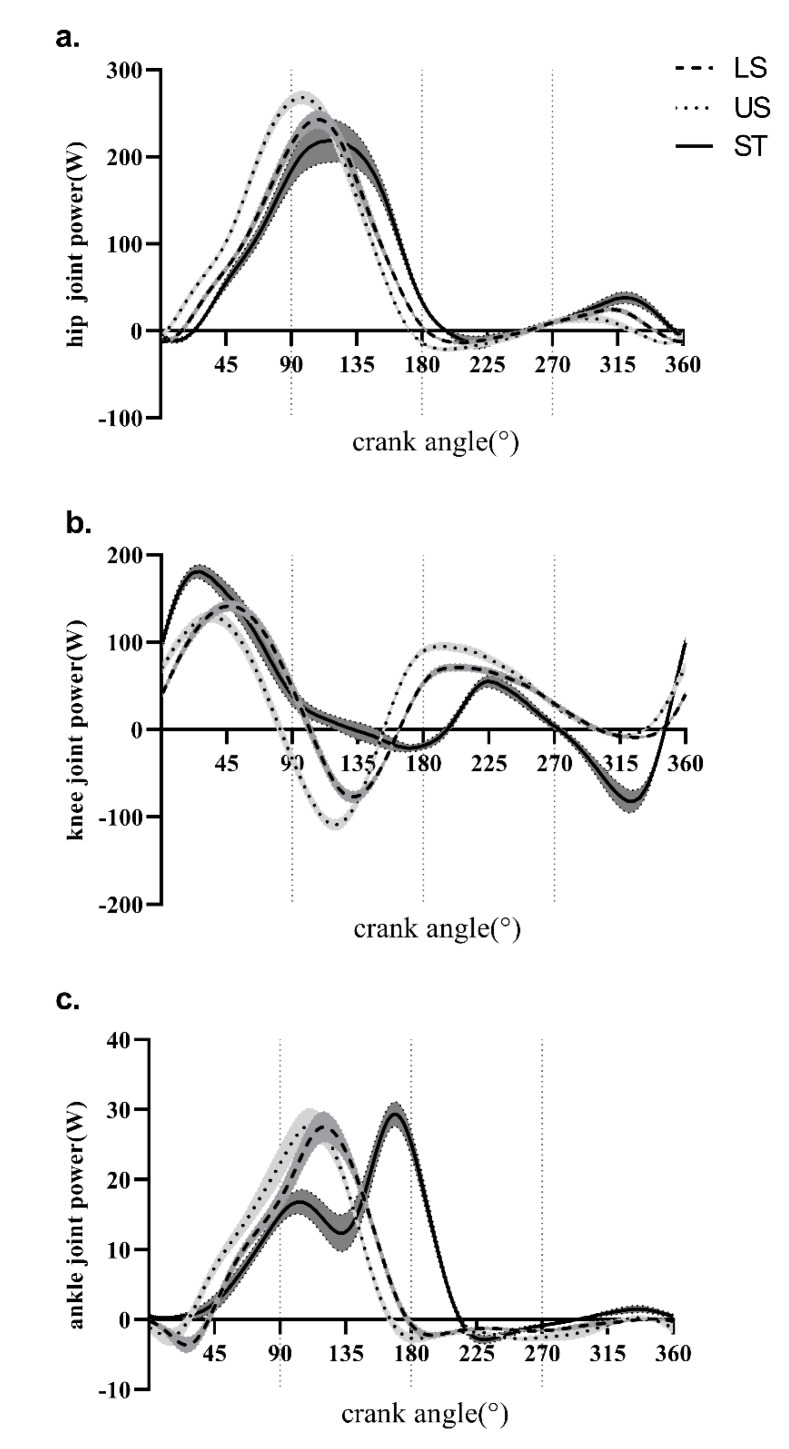
Average curves of (**a**) hip, (**b**) knee, and (**c**) ankle joint power (±SEM) during cycling in the LS, US and ST conditions at a constant power output. (**a**) hip joint power; (**b**) Knee joint power; (**c**) ankle joint power; The vertical dash line in 90°, 180° and 270° refers to the border between each adjacent phase from P1 to P4.

There was a significant difference in joint moments between US and ST conditions in the hip joint (P1: *p* < 0.0001; P4: *p* = 0.0012), knee joint (P1: *p* = 0.0056; P2: *p* < 0.0001; P3: *p* = 0.0142; P4: *p* = 0.0005), and ankle joint (P1 and P2 *p* < 0.0001; P3: *p* = 0.0013; P4: *p* = 0.0003). The hip extension moment in the ST condition decreased in P1 compared to the US condition. However, the hip moment changed from extension to flexion when the seat position changed from the US to the ST condition in P4 (*p* = 0.0012). Compared to the US condition, the knee joint in ST showed an increased extension moment in P1 and P4 (*p* < 0.05), and a reduced flexion moment in P2 and P3 (*p* < 0.0001, Figure 3b). The ankle joint in the ST condition showed an increased plantarflexion moment in P2 and P3, and a decreased plantarflexion moment in P1 compared to the US condition, while the ankle joint moment changed from plantarflexion to dorsiflexion in P4 in the ST condition.

The results showed that the crank moment was only affected by the slope in P1 (*p* = 0.036). However, no significant difference was observed in the crank moment between the LS and US conditions from P2 to P4. Besides, the crank moment was significantly affected by body postures in P2 and P4 (*p* < 0.05). No significant difference was observed in the crank moment between the US and ST conditions in P1 and P3. 

### 3.2. Joint Mechanical Work

Average profiles of the hip, knee, and ankle power (±SEM) in the LS, US and ST conditions are outlined in Figure 3.

The joint mechanical work of the ankle, knee, and hip joints to TMW in LS, US, and ST conditions from P1 to P4 and one crank cycle are summarized in Table 2.

In one crank cycle, there were no significant effects of slope and posture on the total mechanical work (TMW), the mechanical work of the hip, knee, and ankle joints, and the relative contribution of each joint to the TMW (*p* > 0.05). However, if each phase is analyzed, the effects of slope and posture on the relative contribution of each joint to TMW are entirely different from those in one crank cycle. On the one hand, considering the slope effects, the contribution of the hip and ankle joint to TMW increased, while that of the knee joint decreased in the US condition compared to the LS condition in P1 (*p* < 0.05). On the other hand, in terms of posture effects, the results showed that the contribution of the hip joint decreased, while that of the knee joint increased in the ST condition compared to the US condition in P1 (*p* < 0.05). Besides, as there was both negative and positive mechanical work by each joint from P2 to P4, the relative contribution of each joint to TMW was not reported.

## 4. Discussion

The primary purpose of this study was to investigate the effects of surface slopes (level seated vs. uphill seated) and body postures (uphill seated vs. uphill standing) on lower extremity joint kinetics, with a cycling model from a biological perspective. One crank cycle was divided into four phases in this study. With this phase division method, more detailed information about joint moments and mechanical work in different cycling conditions was acquired. The results were shown in the moment–crank angle profiles (Figure 2) and the power–crank angle profiles (Figure 3) in the LS, US and ST conditions.

Our first hypothesis was that the surface slope would significantly alter the lower extremity joint moments and mechanical work, which was partially supported by the results of this study. Variances in slopes from level ground to a 14% slope significantly changed the lower extremity joint moments and mechanical work in some phases. The uphill seated condition exhibited an increased hip extension moment and ankle plantarflexion moments, a decreased knee extension moment in the early downstroke phase (P1), and these peak moments shifted slightly earlier in the crank cycle. These results are consistent with Caldwell et al. [30,34], who reported similar joint moment profiles for different slopes. From the perspective of joint mechanical work, there were no significant changes in mechanical work in the hip, knee, or ankle joints and the total mechanical work (TMW), when the ground changed from level to a 14% slope, which is consistent with findings in Bini et al. [45] and Ericsson [46]. However, the relative mechanical work contribution of the hip joint to TMW in the US condition was significantly increased compared to the LS condition in P1. In other words, the hip joint was the dominant joint in P1 during uphill cycling. This may be due to the increase of the hip joint flexion angle as the ground changed from level to a 14% slope during cycling. Meanwhile, the length of the hip extensor increased with the hip flexion angle increasing, and the increase in the length of the hip extensor muscles has been suggested as an attempt to maximize muscle power production [47]. If this was the case, the hip joint extensors might produce more power, with the hip flexion angle increasing during uphill cycling. 

The second hypothesis was that the lower extremity joint function would change with the varying body postures from seated to standing during cycling, which was also supported by the results from the analysis of joint moments and mechanical work in this study. On the one hand, considering mean joint moments from P1 to P4, the hip joint moment function changed from extension to flexion, with postures changing in P4. Meanwhile, the ankle joint moment function changed from plantarflexion to dorsiflexion with postures changing in P4. However, the functions of the knee joint moment and the crank moment were not altered, with postures varying from seated to standing during cycling. Our results are consistent with the results of Caldwell et al. [30], who found that the hip joint moment profile exhibited a decreased and later peak extension moment from US to ST conditions. The peak of ankle plantarflexion moment showed a significant increase and shifted later when comparing ST with US conditions. The peak knee joint flexion moment shifted significantly during the late downstroke period. Changing from uphill seated to uphill standing delayed the onset of the peak knee joint flexion moment and resulted in a reduced flexion moment magnitude and duration. On the other hand, considering the mechanical work of each joint, similar results were also observed from P1 to P4. It is a standard method to integrate the mechanical power in one crank cycle to calculate the mechanical work during cycling [37,38]. In this way, there was no significant difference between US and ST conditions in mechanical work in one crank circle. However, when the relative mechanical work contribution of each joint was analyzed in each phase, it was found that the power resource joint in the ST condition was the knee joint rather than the hip joint in P1, and the power resource joint was the hip joint in the US condition in P1 and P2. Therefore, it was found that the postural change from seated to standing may change the work pattern of lower extremity joints during cycling, and, especially, may increase the work contribution of the knee joints in P1. The change of postures meant that the hip joint was not the only source of power. In the ST condition, the magnitude of the knee joint extension moment was more than in the US condition. Therefore, it indicated that the knee joints also played a role as a power source.

Undoubtedly, altering postures from seated to standing dramatically changed the joint moments and pedal force, due to changes in the geometry of body segments and removal of the saddle as a support for the cyclists during cycling [7]. The resultant joint moments must balance gravitational, inertial and external force effects. However, the magnitude and direction of the pedal force are major determinants of lower extremity joint moments [48]. Uphill cycling in a standing posture results in an increased contribution of gravitational forces to pedal force during the downstroke phase, due to the loss of saddle-support [34]. Caldwell et al. pointed out that the knee extension moment could be used throughout the entire downstroke during uphill standing cycling [30]. However, our results do not support their conclusion. Since extra gravitational force should be delivered to the pedal, the knee joint absorbs energy from the hip joint and transfers power to the pedal. That could explain why the knee extension moment was larger in the ST condition than in the US condition from P1 to P4. Similarly, the ankle joint moment in the ST condition showed a larger plantarflexion moment than in the US condition.

The main limitation of this study was that the tests were performed in the lab setting rather than under real road conditions. Besides, the participants in our study were not elite cyclists. Thus, it remains unknown whether our conclusions are also valid for top-level cyclists, which might be a potential limitation of this study. Lastly, the inertia force was not cut off in the data reduction, which might overestimate lower limb joint moment and mechanical work. However, the inertia force is less than 5 N in this study, so its influence on the joint moments and mechanical work was minimal.

## 5. Conclusions

In summary, it can be concluded that both surface slope and body posture influence the lower extremity joint kinetics during cycling. Besides hip joints, the knee joints also played the role of a power source during uphill standing cycling in the early downstroke phase, and in this case, the load of the knee joint increased at this time. It is recommended to adopt a standing posture for more power output during uphill cycling, but not for long periods, in view of the risk of knee injury. Future studies could further explore the relationship between standing posture and knee injury during uphill cycling, in order to guide cycling training.

## Figures and Tables

**Table 1:** Mean moments (±SD) of the hip, knee, and ankle joints, and the crank in LS, US, and ST conditions from P1 to P4 at a constant power output (N·m).

Joint	Phase	LS	US	ST
Hip	P1	57.03 ± 10.37	70.33 ± 12.55 *	39.94 ± 11.87 ^#^
	P2	68.01 ± 10.92	70.46 ± 9.26	66.17 ± 14.59
	P3	9.81 ± 6.97	10.95 ± 7.27	10.33 ± 9.42
	P4	−3.15 ± 4.86	2.89 ± 6.17 *	−9.86 ± 7.29 ^#^
Knee	P1	31.63 ± 9.54	21.84 ± 9.41 *	31.25 ± 9.3 ^#^
	P2	−20.16 ± 9.29	−31.59 ± 7.18 *	−2.84 ± 11.82 ^#^
	P3	−16.71 ± 3.67	−18.56 ± 3.87	−11.5 ± 5.81 ^#^
	P4	5.6 ± 5.22	7.41 ± 6.02	21.49 ± 9.68 ^#^
Ankle	P1	20.02 ± 4.81	26.39 ± 5.9 *	14.49 ± 3.79 ^#^
	P2	33.3 ± 3.81	33.34 ± 4.25	50.77 ± 9.81 ^#^
	P3	5.94 ± 1.97	7.29 ± 2.38 *	11.98 ± 2.75 ^#^
	P4	1.69 ± 1.67	3.42 ± 2.73 *	−1.24±1.85 ^#^
Crank	P1	36.46 ± 5.09	36.64 ± 6.08	33.37 ± 5.82
	P2	39.47 ± 3.26	39.99 ± 5.37	58.19 ± 8.85 ^#^
	P3	−0.18 ± 1.81	−1.82 ± 2.51 *	−2.27 ± 8.26 ^#^
	P4	−2.23 ± 2.05	−3.01 ± 3.23	−1.56 ± 3.45 ^#^

(*) Significant differences were noted between LS and US conditions; (^#^) Significant differences were noted between US and ST conditions; P1: 0° to 90°; P2: 90° to 180°; P3: 180° to 270°; P4: 270° to 360°.

**Table 2:** Average ± SD results of the mechanical work of the ankle, knee and hip joints in the LS, US, and ST conditions from P1 to P4 and one crank cycle at a constant power output.

Joint	Phase	Mechanical Work (J)			Relative Mechanical Work (%)		
		LS	US	ST	LS	US	ST
Hip	P1	19.4 ± 4.2	28.7 ± 4.9 *	14.5 ± 5.0 ^#^	44.9 ± 11.9	60.4 ± 13.4 *	32.6 ± 10.7 ^#^
	P2	36.3 ± 8.2	33.8 ± 6.3	40.8 ± 13.6 ^#^	-	-	-
	P3	−1.4 ± 1.8	−2.6 ± 2.3 *	−0.4 ± 2.5 ^#^	-	-	-
	P4	2.1 ± 2.4	0.3 ± 3.0	5.3 ± 3.7 ^#^	-	-	-
	one cycle	56.4 ± 11.0	60.2 ± 8.2	60.1 ± 18.7	60.9 ± 11.5	65.0 ± 12.0	63.4 ± 11.6
Knee	P1	23.5 ± 6.9	17.6 ± 8.4 *	29.5 ± 7.9 ^#^	53.0 ± 12.5	36.1 ± 13.9 *	64.9 ± 10.9 ^#^
	P2	−7.1 ± 6.3	−8.9 ± 4.3	−1.5 ± 6.8 ^#^	-	-	-
	P3	14.3 ± 3.7	17.3 ± 3.5 *	6.2 ± 3.4 ^#^	-	-	-
	P4	1.4 ± 1.6	2.9 ± 1.2 *	−6.6 ± 5.1 ^#^	-	-	-
	one cycle	32.0 ± 11.5	28.9 ± 12.5	27.6 ± 11.1	34.4 ± 11.7	30.3 ± 12.0	29.9 ± 11.6
Ankle	P1	0.9 ± 0.6	1.7 ± 0.9 *	1.0 ± 0.6 ^#^	2.2 ± 1.4	3.5 ± 1.9 *	2.6 ± 1.2
	P2	3.9 ± 1.3	3.4 ± 1.0	4.3 ± 1.2	-	-	-
	P3	−0.4 ± 0.2	−0.5 ± 0.2 *	0.6 ± 0.5 ^#^	-	-	-
	P4	−0.1 ± 0.2	−0.2 ± 0.2	0.1 ± 0.3 ^#^	-	-	-
	one cycle	4.3 ± 1.6	4.3 ± 1.6	6.1 ± 1.2	4.8 ± 1.9	4.7 ± 1.8	6.7 ± 1.7
TMW	one cycle	92.8 ± 5.8	93.4 ± 7.0	93.9 ± 17.4	-	-	-

LS: level seated; US: uphill seated; ST: uphill standing; (*) significant differences between LS and US; (^#^) significant differences between US and ST; P1: 0° to 90°; P2: 90° to 180°; P3: 180° to 270°; P4: 270° to 360°; (-) not available.
